# Natural Plant-Derived Chemical Compounds as *Listeria monocytogenes* Inhibitors In Vitro and in Food Model Systems

**DOI:** 10.3390/pathogens10010012

**Published:** 2020-12-25

**Authors:** Iwona Kawacka, Agnieszka Olejnik-Schmidt, Marcin Schmidt, Anna Sip

**Affiliations:** Department of Food Biotechnology and Microbiology, Poznan University of Life Sciences, Wojska Polskiego 48, 60-627 Poznan, Poland; iwona.kawacka@up.poznan.pl (I.K.); marcin.schmidt@up.poznan.pl (M.S.); anna.sip@up.poznan.pl (A.S.)

**Keywords:** natural antimicrobials, antibacterial plant compounds, *Listeria monocytogenes*, food quality, safety, essential oils

## Abstract

*Listeria monocytogenes* is a foodborne pathogen, sporadically present in various food product groups. An illness caused by the pathogen, named listeriosis, has high fatality rates. Even though *L. monocytogenes* is resistant to many environmental factors, e.g., low temperatures, low pH and high salinity, it is susceptible to various natural plant-derived antimicrobials (NPDA), including thymol, carvacrol, eugenol, *trans*-cinnamaldehyde, carvone S, linalool, citral, (E)-2-hexenal and many others. This review focuses on identifying NPDAs active against *L. monocytogenes* and their mechanisms of action against the pathogen, as well as on studies that showed antimicrobial action of the compounds against the pathogen in food model systems. Synergistic action of NDPA with other factors, biofilm inhibition and alternative delivery systems (encapsulation and active films) of the compounds tested against *L. monocytogenes* are also summarized briefly.

## 1. Listeria Monocytogenes

*Listeria monocytogenes* is a foodborne pathogen, causative agent of listeriosis, which is a disease with high hospitalization and fatality rates, especially in pregnant women, newborns, the elderly and immune-compromised individuals [1,2,3]. Once ingested, the pathogen is able to cross the intestinal barrier, disseminating to the spleen and liver. It can also cross the blood-brain barrier, leading to meningitis, or the placental barrier, causing miscarriages and stillbirths of the fetus [1,3,4,5]. Many virulence factors were determined for *L. monocytogenes*, out of which listerial hemolisin, called also listeriolysin O (LLO) was the first one [5]

The pathogen can be found in various environments, such as soil, manure and water [6] and in variety of raw foods, such as uncooked meats and vegetables, as well as in ready-to-eat (RTE) foods, contaminated during or after processing [1,6]. *L. monocytogenes* can survive and proliferate over a wide range of environmental conditions, including refrigeration temperatures, low pH and in high salt concentrations, what makes it especially hard to eliminate from food products [2]. The ability to form biofilms is also a crucial factor in the survival of *L. monocytogenes*. Biofilms are composed of numerous cells attached to a biotic or an abiotic surface and to each other, surrounded by an extracellular matrix predominantly made up of polysaccharide material, but containing also proteins and extracellular DNA [2,7]. Presence of the pathogen in food is strictly regulated by legal authorities and in many countries, including European Union. Either complete absence of the pathogen in food product is required or the pathogen may be present, but it may not exceed 100 CFU/g (colony forming units per gram) throughout the shelf-life, depending on the food product properties. In the United States, for example, so-called “zero tolerance policy” requires absence of the pathogen in all food products. Complying with the legal microbiological criteria is still a great challenge for food manufacturers [8,9]. Natural plant-derived antimicrobials (NPDA) are promising tools, which can help to mitigate *L. monocytogenes* in food products.

## 2. Antilisterial Plant-Derived Compounds

Various natural plant-derived substances and chemical compounds have antibacterial effect [10,11,12], including essential oils and their components [13,14,15,16]. Essential oils (EOs) (also called volatile or ethereal oils) are aromatic oily liquids obtained from plant material (flowers, buds, seeds, leaves, twigs, bark, herbs, wood, fruits and roots) [13,17,18]. They are poorly soluble in water but highly soluble in alcohol, organic solvents, and fixed oils [17,19]. Essential oils exhibit a very characteristic fragrance and are therefore responsible for the specific scents that aromatic plants emit [19]. EOs are complex mixtures that contain 2 or 3 main components at a level of 20–70% up to 90%, however EOs may contain over 300 different compounds [17,19,20], even up to over 500 in some oils [21]. Plants produce many secondary metabolites, namely terpenoids, aldehydes (e.g., citral, benzaldehyde, cinnamaldehyde, carvonecamphor), alcoholic compounds (e.g., geraniol, menthol, linalool), ketonicbodies (e.g., thymol, eugenol), acidic compounds (e.g., benzoic, cinnamic, myristic acids), and phenols (e.g., ascaridole, anethole). Among those, terpenes (e.g., terpinene, pinene, myrcene, limonene, p-cymene) and terpenoids (e.g., oxygen-containing hydrocarbons) are classified as aromatic phenols (e.g., thymol, carvacrol, safrole, eugenol) and have major roles in the composition of various essential oils [15]. Additionally to their antibacterial action, essential oils were proven to have antiviral, antimycotic, antiparasitic, antioxidant and insecticidal activity [18].

Many EOs present activity against *L. monocytogenes*, for example: gardenia, cedarwood, bay leaf, clove bud, oregano, cinnamon, allspice, thyme, patchouli, white camphor, lemon verbena, angelica root, cassia, cinnamon leaf, clove leaf and clove bud, basil, bergamot, pimento, bay, *Eucalyptus radiata* and citriodora, tea-tree and lemongrass oils [22,23]. Many of them come from spices known for centuries and widely used for seasoning food products. EOs have also been successfully used for reduction of *L. monocytogenes* not only in vitro, but also in various food model systems (including meat, milk, vegetables and fruits) as published by many authors even in recent years [17,24,25,26,27,28,29,30,31,32,33,34,35]. Not only EOs are antilisterial, but also various extracts, for example grape extracts (skin, seed, stems, pomace, juice and berries extracts) [36,37,38], blueberries extracts [37], lowbush berries extracts [39], apple skin, olive pomace, olive juice powder extracts [40] and grapefruit seed extract [41], as well as *Achillea schurii* flowers extracts [42] showed activity against *L. monocytogenes*. The wide selection of NPDAs brings a great opportunity to mitigate *Listeria* sp. in a variety of food products, not only meat based, but also vegetable, fruit, as well as sweets and desserts. Moreover, NPDAs open a path for designing innovative food products with clean label and novel fragrance, which would meet expectations of the demanding customer.

This review however focuses on plant-derived compounds (particular chemical substances) with proven antilisterial activity and not the EOs or extracts themselves. The composition and consequently the activity of the EOs or extracts may significantly vary within the “same” oils (i.e., originating from plants of the same species, but obtained from different suppliers) [43] or depending on the solvent used for extraction (e.g., cinnamon extracts extracted with N-butane showed much higher antilisterial activity than ethanol extracts) [44]. Thus, experiments with purified compounds are more replicable and what is of major importance when optimizing conditions of eliminating the food pathogens.

One of the first studies aiming to determine antilisterial compounds in EOs was performed by Lis-Balchin and Deans [43]. In the study, 93 different commercial EOs were screened for activity against 20 *L. monocytogenes* strains in vitro by agar wells diffusion method. Results of the experiment suggested a probable correlation between strong antilisterial activity and the main components of these EOs: eugenol, cinnamaldehyde, thymol, citral, geraniol (naturally occurring in rose [22]), citronellol (naturally occurring in geranium [45]), limonene (occurring in lemon [22]) and other monoterpenes. Examples of plant sources of the compounds most often mentioned throughout this publication are listed in Table 1.

In latter study performed by Friedman et al. [22], 96 EOs and 23 oil compounds were tested against two *L. monocytogenes* strains. However, the authors stated that standard diffusion methods based on measurement of inhibition zone of bacterial growth on solid medium is not accurate for EOs, as zones of inhibition may vary, depending on the ability of oil to diffuse through an agar medium and also vapors of the oil may influence bacterial growth. Thus, according to authors, the method is not suitable for comparing bactericidal activities of EOs and their compounds. Authors used a microtiter plate bactericidal assay and determined bactericidal activity, which was defined as the percentage of the sample in the assay mixture that resulted in a 50% decrease in colony forming units relative to a buffer control (BA50). According to the authors, the oil compounds that were most active against *L. monocytogenes* were cinnamaldehyde, eugenol, thymol, carvacrol, citral, geraniol, perillaldehyde (naturally occurring in mandarin peel), carvone S, estragole (naturally occurring in tarragon), and salicylaldehyde (naturally occurring in almonds). Substances with BA50 values lower than 0.5% (for at least one strain tested) are listed in Table 2. In other study also BA50 values of epigallocatechin gallate (naturally occurring in green tea) and oleuropein (naturally occurring in olives) were determined against *L. monocytogenes* RM2199 and resulted with 1.42% and 1.43%, respectively [40].

Bagheri et al. [51] made an attempt to create a mathematical model of minimal inhibitory concentrations (MIC) of essential oils against various microorganisms (including *L. monocytogenes*), depending of the chemical composition of the oils. The authors tested 38 essential oils, determined their chemical composition and inhibitory activity against microorganisms. The most significant compounds of essential oils affecting the inhibition of *L. monocytogenes* LMA1045 were phenols and aldehydes, although sesquiterpenes and esters are also included in the created model. Created linear model is statistically significant (with the *F* value of 5.8 and *p* value of 0.0012) and is as follows: MIC of *L. monocytogenes* LMA1045 = 15,989.3 − 13.8*Sesquiterpene − 15.3*Aldehyde − 13.0*Phenol − 30.6*Esters.

Minimal inhibitory concentrations and minimal bactericidal concentrations (MBC) for natural antilisterial compounds are listed in Table 3 and Table 4, respectively. However, MIC or MBC values of the compounds cannot be directly compared within different references, as experiment conditions, e.g., temperatures of incubation, chosen medium or pathogen strain, as well as method used for determination of inhibitory or bactericidal concentration may influence the inhibitory or bactericidal values. What is more, MIC or MBC against *L. monocytogenes* has not always been determined for natural compounds, even though the components are proven to be antilisterial (usually by creating zone of inhibition on agar medium during diffusion tests). For example cymene (naturally present in oregano and thyme) [52], berberine [53], S-carvone, decanal [54], iberin, erucin, sulforaphane, benzylisothiocyanate and 2-phenylethylisothiocyanate [55], quercetin [36], also many acid compounds, i.e., rosmaric acid, caffeic acid (both naturally occurring in *Melissa officinalis*), hydroxyphenyllactic and *trans*-cinnamic acids [56], p-coumaric acid [36,56], gallic acid, ferulic acid [36], as well as peptides derived from rice or palm kernel cake [11] are antilisterial, however their MIC or MBC values against *L. monocytogenes* remain undetermined.

## 3. Mechanisms of Action

Only some of the substances known to be inhibitory against *L. monocytogenes* have been determined for their exact mechanism of action against the pathogen. However, some of the experiments were performed with *Listeria innocua*, which is closely related to *L. monocytogenes*, though not pathogenic to humans [82,83]. Traditionally, *L. innocua* have been used as a model for predicting *L. monocytogenes* behavior in food processing environments, as the two microorganisms share the ecological cohabitation and are similar physiologically. Existing differences between the two species and results obtained by examination of one of them may not be prudent to the other [83]. Similarly, as an antibiotic resistance differ for various strains of the same species, the same phenomenon can to be expected for NPDA susceptibility. Therefore, screening of numerous strains can provide truly informative overview. Nonetheless, Silva-Angulo et al. [84], after their experiments comparing the growth and injury of the two bacteria caused by citral, suggested that *L. innocua* could be used as surrogate for *L. monocytogenes* when testing the effect of the antimicrobial, as *L. innocua* due to its shorter lag phase, represents a worst case scenario. However, the mechanisms of action of most of the antilisterial compounds has not been examined for neither *L. monocytogenes*, nor *L. innocua*, as authors focused mostly on mechanisms of action of carvacrol, eugenol, cinnamaldehyde, thymol and citral.

One of the first studies aiming to determine mechanisms of the NPDA action against *L. monocytogenes* was performed with eugenol and cinnamaldehyde. Presence of the compounds prevented an increase in the cellular ATP levels upon glucose addition in nonenergized cells. Authors concluded, that effects of energy generation are crucial at bactericidal concentrations of eugenol and cinnamaldehyde [81]. During further experiments on isolated *L. monocytogenes* membranes, the membrane bound ATPase activity was significantly inhibited by eugenol (5 or 10 mM), carvacrol (10 mM) and cinnamaldehyde (10 mM) [85]. In other study the group focused on cell membrane disruption caused by the plant EO aromatic compounds. Eugenol and carvacrol, unlike cinnamaldehyde (up to 10 mM), increased uptake of propidium iodide by *L. monocytogenes* [86]. Increased propidium iodide internalization is an indicator of large pore formation, usually leading to the cell death [87]. All three compounds inhibited *L. monocytogenes* motility, as well as caused rapid decrease of the cellular ATP. Eugenol and carvacrol caused release of ATP. Experiments indicated that the primary mechanism of action of eugenol and carvacrol at bactericidal concentration is disruption of the cell membrane, leading to increased non-specific permeability, whereas in cinnamaldehyde evidence for membrane disruption was less evident [86]. However, in other studies, the transmission electron microscopy of the *L. monocytogenes* ATCC 13932 cells after treatment with cinnamaldehyde (0.1%) at 37 °C for 6 min, revealed that the compound damages cell membrane, leading to disruption of its integrity and leakage of the cytoplasmic content [88]. Presence of *trans*-cinnamaldehyde caused perturbations in the cytoplasmic membrane fluidity, potential and intracellular pH in *L. innocua* LRGIA01. As hypothesized by the authors the changes happened due to accumulation of the *trans*-cinnamaldehyde in the hydrophobic core of the cytoplasmic membrane of the cells. However, similarly to the Gill and Holly [86] results, the membrane changes did not increase propidium iodide uptake, suggesting that cells survived the treatment and entered viable but not culturable state (VBNC) [87]. Flow cytometric assessment showed that the membrane permeabilization occurred when *L. monocytogenes* Scott A was exposed to the sub-inhibitory and MIC values of citral, carvacrol and (E)-2-hexenal. Esterase activity remained unchanged after treatments [89].

Sub-lethal concentrations of carvacrol, thymol and citral caused changes in the membrane fatty acid composition of *L. monocytogenes* Scott A, depending on the compound and its concentration. Carvacrol caused an increase in general unsaturation level and chain length of the membrane fatty acid composition, citral mainly induced an increase of unstaturation level, whereas in thymol changes in the parameters were less evident, however for example C 18:1*trans*9 concentration in the presence of 100 ppm of thymol increased from 2.1% (control sample) to 16% [90]. Similarly, Rogiers et al. [91] concluded that the membrane fatty acid composition is a determinant of *L. monocytogenes* sensitivity or resistance to *trans*-cinnamaldehyde, as *L. monocytogenes* mutant lacking in *IlvE* gene was hypersensitive to the antimicrobial. IlvE encoded by the gene is a branched-chain amino acid aminotransferase, that reversibly catalyzes the transamination of the branched-chain amino acids to the corresponding short-chain α-ketoacids. Whole-cell fatty acid composition of the hypersensitive mutant revealed significant reduction of anteiso branched-chain fatty acids which were replaced by unbranched saturated fatty acids and iso-branched-chain fatty acids.

Cell membrane composition changes may also be a factor determining temperature dependence of the activity of the antimicrobials. Usually at higher temperature, the compounds exhibit higher antimicrobial properties, for example reduction of *L. monocytogenes* achieved with 3 mmol/L carvacrol reached 0.6 log units at 1 °C and 1.8 log units at 20 °C [92], and in other study the highest activity of carvacrol was observed at 30 °C (compared to 10 °C and 20 °C) [63]. Karatzas et al. [54] performed an experiment, where exponential phase cultures grown at 8 °C, 35 °C or 45 °C were subjected S-carvone at 45 °C for 30 min. Only cells pre-grown at 8 °C showed reduction in viable counts. Authors concluded that changes in phospholipid composition of the cytoplasmic membrane may be the reason for the phenomenon. Cells pregrown at lower temperatures maintain membrane fluidity by changes in fatty acid composition and if these cells are transferred to higher temperatures, fluidity of their membranes is higher than would be if the cells had been cultured at higher temperature. As a consequence, S-carvone dissolves into lipid bilayer of the cells pregrown in lower temperatures in higher amounts. However, cells from stationary phase were not susceptible to S-carvone at 45 °C, whether they were pregrown at 8 °C or 45 °C. Authors suggested that this phenomenon is due to the physiological changes such as decreased membrane fluidity and permeability and synthesis of stress proteins, as bacteria undergo these type of changes when they enter stationary phase [54].

### 3.1. Gene Expression Responses and Proteome Changes

Plant-derived substances present in the environment influence level of gene expression of *L. monocytogenes* cells. Consequently, they also influence bacterial proteome.

*Trans*-cinnamaldehyde, carvacrol and thymol at the sub-inhibitory and inhibitory concentrations statistically significantly down-regulated *L. monocytogenes* virulence genes (including among others internalins, listeriolysin O, phospholipases, actin polymerization protein as well as proteins associated with motility) expression levels in all three strains of the bacteria that were used in the study (ATCC 19115, Scott A and Presque-598) [59]. Down regulation of virulence factors of the same three strains of *L. monocytogenes* was also confirmed for eugenol [93].

*L. monocytogenes* exposed to sub-inhibitory concentrations of *trans*-cinnamaldehyde, carvacrol, thymol and eugenol showed changes in the expression of 13 (out of 13 targeted) critical biofilm associated genes, namely flagellar proteins, transcriptional regulators and quorum sensing genes. Relative fold change in the expression of these genes was negative in all cases, suggesting clear down-regulation. However, in two and three genes encoding flagellar proteins in *trans*-cinnamaldedyhe and eugenol treatment, respectively, the fold change was not significantly different [79].

Relative gene expression analysis was also performed with a sub-inhibitory concentrations of (E)-2-hexenal, citral, carvarcol on *L. monocytogenes* Scott A. Targeted genes selected in the study were responsible for various cell functions, including cell division and DNA repression and modulation, catabolic processes, synthesis of RNA and proteins, stress response and virulence and motility. Citral and carvacrol showed the same mechanism of action, different from (E)-2-hexenal. Citral and carvacrol caused an overexpression of *cpsL* and *bsh* genes, involved in stress response and motility, respectively. (E)-2-hexenal caused imbalance in catabolic processes, interpreted as a hypothetical change from oxidation to fermentation, executed by down-regulation of *pgm* and *pdhD* involved in glycolysis. (E)-2-hexenal was inhibitory towards the expression of genes responsible for stress response, protein synthesis and DNA protection and repair [58].

Thymoquinone at concentration of 1.56 μg/mL (whereas MIC was of 12.5 μg/mL) significantly down-regulated expression level of *L. monocytogenes* ATCC 19115 all seven targeted genes associated with motility, biofilm formation, hemolysin secretion, and adhesion and invasion of the host cells. A concentration of 0.78 μg/mL also caused down-regulation of all analysed genes, however in two cases the decrease was not significant [77].

*L. monocytogenes* Scott A treated with sub-MIC concentrations of citral, carvacrol and (E)-2-hexenal showed increased or decreased (≥ or ≤2-fold) levels of 223 protein spots (compared to profile of cells grown under optimal conditions), which affect many major cellular processes, including cell cycle control, cell division, chromosome, motility and regulatory related proteins, metabolism of carbohydrates, pyruvate, nucleotides and nitrogen, vitamins cofactors and also stress response proteins. Citral and (E)-2-hexenal adapted cells increased survival under acid stress [94].

Presence of carvacrol in tyrosine decarboxylase broth significantly changed production of the biogenic amines and ammonia by *L. monocytogenes* ATCC7677. Carvacrol at all 3 tested concentrations (0.1%, 0.5% and 1%) when compared to the control reduced production of: ammonia, putrescine, 2-phenyl-ethylamine, spermine, tyramine and agmatine. Interestingly, higher dose not always corresponded with greater reduction, e.g., ammonia and putrescine production were higher at 1% carvacrol than at 0.5% carvacrol. Production of caraverine, spermidine, tryptamine, histamine and dopamine were increased by 0.1% carvacrol as compared to the control, however reduced compared to the control at higher concentrations (0.5% or 1%). Trimethylamine production was reduced by 0.1% carvacrol and 0.5%, however 1% caused increased production. Serotonin was the only biogenic amine tested that lowest levels of production were achieved in the absence of carvacrol [95].

### 3.2. Bacterial Adaptation

Apolónio et al. [68] evaluated the impact of continuous exposure of three *L. monocytogenes* strains (Scott A, EGD, C882) to eugenol and citral. To determine ability of the pathogen to gain resistance to these compounds, the bacterial strains were subjected to sequential passages in the presence of sub-inhibitory to lethal concentrations. Following the four sequential passages at the desired concentration of the antilisterial compound, bacteria were transferred to the medium with higher concentration of the compound. The process was repeated until bacteria stopped growing. The *L. monocytogenes* EGD and Scott A adapted cells were not able to overcome the first passage at the MIC concentration of eugenol (after four passages at sub-inhibitory concentration). The *L. monocytogenes* C882 adapted cells overcame four passages at sub-inhibitory and MIC concentration but failed to overcome the first passage at the MBC concentration of eugenol. Identically, the *L. monocytogenes* EGD and C882 adapted cells overcame the four passages at MIC concentration of citral, but failed the first passage at MBC concentration. The *L. monocytogenes* Scott A adapted cells were only able to overcome three passages at MBC concentration of citral. In conclusion, none of the strains was able to mount a resistance response after the adaptation exposure to sub-inhibitory concentrations of both compounds. Similarly, in other experiment *L. monocytogenes* Scott A cells were cultured in presence of S-carvone at sub-inhibitory concentration and further combined treatment with mild heat was equally effective in adapted and unadapted cells [54].

Influence of the adaptation to eugenol and citral at their sub-inhibitory concentrations (0.05 mg/mL) on the susceptibility to 5 antibiotics (penicillin, chloramphenicol, kanamycin, vancomycin and erythromycin) was also evaluated. The sequential passages of all tested strains (*L. monocytogenes*—EGD, Scott A and C882) to eugenol or citral did not induce resistance to the antibiotics tested [68].

### 3.3. Virulence in In Vitro and In Vivo Models

As described earlier, studies are consistent that NPDA down-regulate expression of genes related to bacterial virulence [58,59,77,79,93]. These finding suggest the decreased virulence of *L. monocytogenes* in presence of that compounds, which was verified by actual in vivo and in vitro tests.

*L. monocytogenes* ATCC 19115, Scott A and Presque-598 in the presence of sub-inhibitory concentration or MIC of *trans*-cinnamaldehyde, carvacrol and thymol showed significantly lowered adhesion and invasion of Caco-2 (human enterocyte like) and human brain microvascular endothelial cell lines. Additionally, motility was significantly reduced compared to the untreated controls, as well as hemolysis of 3% sheep red blood cells and phospholipase activity [59]. Similar results were obtained for sub-inhibitory concentration of eugenol, as the antimicrobial reduced hemolysis of 3% sheep red blood cells and adhesion and invasion rate of Caco-2 cells [93]. Similar results were achieved for sub-inhibitory concentrations of thymoquinone present in *L. monocytogenes* ATCC 19115 environment. The treated bacteria showed reduced ability to adhere and invade Caco-2 cells. Thymoquinone at concentrations of 0.39, 0.78, and 1.56 μg/mL (compared to MIC of 12.5 μg/mL), reduced adhesion rate of the cells to 88.36, 80.03, and 74.02% compared to the control, whereas the invasion rate was reduced to 81.59, 52.06, and 46.08%, respectively. Listeriolysin O secretion rates of the pathogen dropped to 42.52, 30.25, and 25.69% in the presence of thymoquinone at concentrations of 0.39, 0.78, and 1.56 μg/mL, respectively [77].

Also in vivo tests determining influence of NPDA on *L. monocytogenes* infection were performed. Injection of 5 log CFU of *L. monocytogenes* per *Galleria mellonella* larvae resulted in 100% mortality by day 5 of the experiment. Pre-treatment with eugenol injection significantly enhanced the survival rates of infected larvae by at least 40% on day 7 [93]. Similar experiment was performed with *trans*-cinnamaldehyde, carvacrol and thymol. All of the compounds increased survival rate of the larvae by at least 20% at day 5. Carvacrol was the most effective and approximately 80% survival rate was observed at day 5 [96].

Apolónio et al. [68] on the other hand determined the impact of adaptation to eugenol on bacterial virulence, using *Galleria mellonella* model. The larvae were injected with two *L. monocytogenes* strains (Scott A or C882) adapted to eugeneol by sequential exposure to increasing concentrations of the compound. Larvae treated with Scott A and C882 adapted cells, survived at significantly higher level than the larvae injected with nonadapted cells along first four days and three days, respectively. No significant differences (*p* < 0.05) were found between survival rates of larvae treated with adapted or nonadapted *L. monocytogenes* at fifth day for larvae treated with C882 and at from fouth to fifth day for larvae treated with Scott A.

Silva et al. [97] evaluated effect of possible virulence changes of *L. monocytogenes* CECT 4032 untreated cells or treated with either citral or carvacrol using *Caenorhabditis elegans* as a model. *C. elegans* showed no loss in life span (*p* < 0.05) when it was fed in a lawn of *L. monocytogenes* previously treated with citral compared untreated *L. monocytogenes*, however the pathogen previously treated with carvacrol showed loss in a life span with last 5 percentile of worms. Within 75, 50 and 25 percentiles differences were not observed. Citral treated *L. monocytogenes* showed no effect on egg laying by *C. elegans*, compared to untreated *L. monocytogenes*, whereas carvacrol treated bacteria reduced statistically significantly the number of eggs.

## 4. Food Model Systems

Efficacy of NPDAs against *L. monocytogenes* was evaluated in various groups of food products. Attempts of inhibiting *L. monocytogenes* inoculated in food products are summarized in Table 5. However, the table focuses only on purified chemical compounds (used either alone or in combination with other substances or methods) and other treatments applied to the contaminated products within the references, even more effective than those incorporating NPDAs, may not be included. Supplementary information to Table 5 (including other, less effective treatments incorporating NPDAs tested in the studies, used bacterial strains and comments) are presented in Table 6. Both tables are connected by the common column with heading “Row”, where in each row a unique two-letter symbol is placed. Corresponding rows within the two tables were marked with identical row symbol.

Even though *L. monocytogenes* reductions were achieved in most of the experiments presented in Table 5, it has been suggested that a 6-log reduction in foodborne pathogens is required for minimally processed foods [52] and reductions in this order of magnitude has only been achieved in some cases. According to strict legal criteria in many countries [8,9] it would be most desirable to achieve complete inhibition of the pathogen in food products or to reduce its presence to the maximum of 100 CFU/g. Notably, in most of the cases of reducing *L. monocytogenes* to undetectable levels NDPA were not used as sole factors. In raw meat combining carvacrol with high hydrostatic pressure (HHP) treatment [98] or immersing meat in teriyaki sauce with thymol [99] resulted in complete pathogen inhibition. The pathogens in teriyaki sauce itself [99] or in soy sauce [101] were inhibited by carvacrol or thymol. In raw fish complete inhibition to undetectable levels was achieved with carvacrol [100]. In meat products, such as frankfurters a successful pathogen eradication was achieved by immersing them in solution containing *trans*-cinnamaldehyde or carvacrol combined with 0.1% hydrogen peroxide along with mild-heat treatment (55–60 °C) for up to 1 min [102]. Additionally, in hams covered with antimicrobial films containing carvacrol [105] or carvacrol or gallic acid paired with chitosan [111] the pathogen was no longer detectable after storage. In milk, although many experiments were performed, only combined treatment with thymol, nisin and lactobionic acid eradicated the pathogen and only in reduced-fat milk [70]. In salad combination of enterocin with either thymol, terpineol or tyrosol [109], in mixed shredded leafy vegetables addition of carvacrol [65] and on cabbage leaves linalool [69] eradicated the pathogen, as well as carvacrol or 1,8-cinole or their combinations in vegetable model broth [65]. On cantaloupes also washing treatments and coating treatments incorporating thymol, carvacrol or caprylic acid combined with hydrogen peroxide eliminated *L. monocytogenes* [110].

Even if the pathogen was not eradicated, successful reduction of the pathogen counts has been achieved in almost all cases. However, in the study of Veldhuizen et al. [63], at end of 4 week storage at 10 °C, contaminated steak tartare treated with 5 mmol/g carvacrol was equally contaminated as the control sample and *L. monocytogenes* counts reached 10^8^ CFU/g. Authors proved that the presence of food components interfered with the activity of carvacrol. Both egg yolk and bovine serum albumin (which are ingredients for tartare steak) inhibited carvacrol activity at >0.2% (wt/vol) in growth medium. Explanation of the phenomenon hypothesized by authors was that that the food product provides more nutrients to bacteria, what leads to quicker cell regeneration. Another explanation is that because EOs and their components are mostly hydrophobic, the antimicrobials are more likely to dissolve in lipid or fat fractions and their access to microorganisms is limited [63,112]. In two experiments comparing antimicrobial action of NPDA in milk, lower reductions were achieved in whole milk compared to reduced fat milk [70,74] and in one case final bacterial counts in whole milk were indifferent from the control sample [74]. In other study the effect of carvacrol combined with HHP was at least two orders of magnitude lower in milk inoculated with *L. monocytogenes* (3.2 log reduction) compared with buffer (>6 log reduction) [92], what leads to conclusion that fat inhibit action of those antimicrobials, as suggested earlier. However even in carrot juice, it was necessary to increase the concentrations of carvacrol and cymene (used in combination) more than 3-times compared to HEPES buffer to achieve desired reduction of *L. monocytogenes* [52]. Additionally, the antimicrobial activity of carvacrol in fresh cheese, chicken breast, fresh-cut pumpkin, and fresh-cut melon and of eugenol in chicken breast and fresh-cut pumpkin against *L. innocua* was less remarkable than in in vitro tests [113]. Concentration of the components not always can be increased in real foods, as the compounds generally reduce the sensory acceptability of food and can be an important limitation for their use as antimicrobial agents [61,112,114]. Many authors suggest that solution for this problem is utilization of the antimicrobials in a system of agents used together in a form of the hurdle technology [81,114]. One author clearly stated that citral and carvacrol can be used as preservatives to control the growth of *L. monocytogenes* only in combinations with other hurdles, as concentrations capable of having lethal effects are incompatible with the food sensory features due to their low sensory threshold [89].

Sensory evaluation of treated samples was performed in only few studies. Sensory evaluation of fillets marinated in solution containing 0.25 mg/mL thymol and 5% (*w*/*v*) salt, showed no significant difference when compared to control sample in both parameters tested, i.e., odor and taste. The maximum on the hedonic scale was 5 (corresponding to most-liked sample) and marinated fillets mean score were of 4.6 and 4.8 for odor and taste, respectively [72]. Soy sauce with up to 1 mM of thymol or carvacrol was scored statistically indifferent of the control soy sauce, reaching approximately 4.5 points on 9 point hedonic scale, where 9 corresponded to the most pleasant odor [101]. Sensory analysis of a shredded leaf mix after treatment with the combination of carvacrol and 1,8-ceineole or the components alone, showed that general perception and taste parameters after 72 h of storage was not significantly different (*p* > 0.05) among all 3 treated samples and the control. In case of the odor parameter after 72 h, only carvacrol sample was different compared to the control and was less desirable. Evaluation was done using 5 point hedonic scale, where the highest score corresponded to like very much [65]. Sensory analysis was also performed on catfish fillets treated with 2% carvacrol. Results indicated, that the aroma compared to the untreated sample is different (*p* < 0.05) in raw and cooked samples. Panelists stated that samples had a menthol or piney aroma in comparison to the control sample, what indicated chemical changes in the compound [100]. Apart from last study, sensory analysis was performed using a hedonic scale of personal preferences. However, ideally, treated product should not only be acceptable or desirable on the same level as untreated product, but it should not be different from untreated product. Thus, antimicrobial treatment would lead not to a final product ready to enter a market, but to a flavor-neutral product base, that could be used to create diverse versions of food products by adding various seasonings and would not limit manufacturers to provide only one flavor version of their product. In one study with *L. innocua*, analysis based on determining if the product is distinguishable from the untreated sample was performed. Addition of 203 ppm carvacrol to turkey breast ham was undetectable by the panelists and thus carvacrol at concentration of 200 ppm was used in the study to determine combined treatment with carvacrol and HHP in turkey breast ham. Complete inhibition of the bacterium was not achieved, as after 60 days of storage at 4 °C the control sample had contamination of approximately 6.5 log CFU/g, whereas treated sample 5.5 log CFU/g [115]. There are no other studies of this kind, where the effect of NPDA applied at a sensory undetectable level would be measured in food products contaminated with *L. monocytogenes* and such experiments would be valuable.

In other experiment, not included in Table 5, impact of activated plastic films with thymol and enterocin AS-48 was evaluated on vacuum-packed sea bream fillets by measuring changes in bacterial load and bacterial diversity under refrigerate storage for 10 days. Samples however were not intentionally inoculated, but natural microflora present was analyzed. Bacteria of *Listeria* genus had the greatest relative abundance (24.90%) of all the microorganisms at day 0 in untreated samples. In samples treated with the activated film, bacteria were reduced to 0.00% of all the microorganisms at day 0. After 10-day storage relative abundance of *Listeria* genus in the treated and untreated samples were of 0.09% and 0.79%, respectively [116].

## 5. Combining NPDA and Other Antilisterial Factors

Synergistic or additive effects are used in food perseveration in the “hurdle technology”, when two or more preserving techniques are used in combination, to achieve gentle but effective preservation of foods. Antimicrobial technologies are used in lower doses to control simultaneously food safety and other food quality aspects such as sensory quality and nutritional values. Synergistic effects may occur among many factors such as pH control, water activity reduction, heat processing, incorporating natural antimicrobials and other [117,118]. Synergism in reducing *L. monocytogenes* between NPDA and other compounds or methods is described later in the text. Some of the experiments were performed with *L. innocua*, which as mentioned above, can be a *L. monocytogenes* surrogate. As suggested by many authors [81,89,114], combined treatment with natural compounds and other methods is necessary for antilisterial treatments of food products.

### 5.1. Plant Antimicrobials Used in Combination

Many compounds showed synergism when they were used in combination. Carvacrol with cymene used together allowed to achieve the same growth delay of *L. monocytogenes* when much lower concentrations of the antimicrobials were used [52]. Carvacrol and 1,8-cineole have also shown synergistic activity against *L. monocytogenes*, which allowed to reduce concentrations of the compounds by 4-folds compared when they were used separately [65]. Fruit extracts (lime, lemon or calamansi) also showed synergism with thymol or carvacrol. When antimicrobials were used in pairs, *L. monocytogenes* counts were reduced by up to 7 log CFU/mL, indicating complete elimination of the pathogen, even though antimicrobials alone were not effective [119]. Binary carvacrol-thymol and thymol-eugenol combinations and ternary combinations of carvacrol, thymol, and eugenol also showed synergistic action against *L. innocua* [114]. Synergy between carvacrol and thymol against *L. innocua* was further confirmed by another author [120]. Combination of monolaurin and *Zataria multiflora* Boiss. essential oil acted synergistically against *L. monocytogenes* under every combination of conditions tested, including 2 levels of temperature (5 °C and 30 °C) and 3 levels of pH (5.0, 6.0 and 7.0) [76]. Ternary combination of natural antimicrobials: grapefruit seed extract, cinnamanaldehyde and nisin showed synergistic effect, reducing *L. monocytogenes* growth on lettuce and on pork loin [41]. Other ternary combination tested against *L. monocytogenes* was carvacrol and thymol in combination with mild heat treatment (55 °C for 30 min). Treatment resulted in reduction of more than 99.99% of *L. monocytogenes* CECT 4031, though the results were statistically indifferent to those obtained when carvacrol or thymol separately were used in combination with heat. However, when shorter heat treatments were applied (5 and 10 min), differences were statistically significant and combination of 3 factors (heat and two antimicrobials together) was more effective than using heat and only one compound [121].

### 5.2. Bacteriocins

Combining plant-derived antimicrobials with bacteriocins, mostly nisin, was thoroughly investigated. Carvacrol and nisin showed synergism at both tested temperatures (8 °C and 20 °C), as *L. monocytogenes* was completely inhibited in the presence of a 16-fold lower concentration of nisin (compared to when nisin was applied as the sole preservative), when it was used along with 1.25 mmol/L of carvacrol [23]. Thymol with nisin Z showed synergy, as 0.02% thymol combined with 40 IU/mL of the nisin Z (even though separately these antimicrobials were ineffective even at higher concentrations) resulted in a great reduction of the growth of *L. monocytogenes*, as indicated by the decrease in optical density value [122]. Nisin A and Nisin V in combination with carvacrol, thymol or *trans*-cinnamaldehyde were more effective, than alone. *L. monocytogenes* was reduced to undetectable levels when 195.2 μg/mL carvacrol was used in combination with 50 μg/mL nisin V, where treatment with these compounds alone resulted in final counts of approximately 7 log CFU/mL and 5.5 log CFU/mL, respectively. Combining Nisin A with EO compound was less effective when compared to nisin V [62]. Combinations of eugenol, thymol, cinnamaldehyde or carvacrol paired with nisin at the concentrations of 1/4 MIC for both antimicrobials used in pairs, resulted in lower final bacterial counts than the control, reaching approximately 7 log CFU/mL for pair with eugenol and thymol, 6 log CFU/mL for pair with cinnamaldehyde and 4 log CFU/mL for pair with carvacrol, compared to 9 log CFU/mL in the control and 6 log CFU/mL as an initial bacterial population [57]. Combining nisin with thymol inhibited *L. monocytogenes* at concentrations 2-fold lower of the two antimicrobials, compared to when used alone. Addition of 1.25 mg/mL of lactobionic acid and creating ternary combination resulted in 4-fold reduction of nisin and thymol [70]. Additionally, binary combinations of eugenol, thymol or carvacrol with nisin or ternary combinations of the three compounds with nisin and diglycerol fatty acid ester, namely diglycerol monolaurate had bactericidal effect on growing *L. monocytogenes* culture, even at concentrations that were ineffective separately [75]. As mentioned earlier, nisin was also incorporated along with cinnamaldehyde and seed extract grapefruit and showed synergism against *L. monocytogenes* cocktail on lettuce and pork loin [41]. In other study however, *trans*-cinnamaldehyde (at concentrations of 0.3 and 0.6 g/kg of curd) showed no synergistic effects with nisin (0.49 g/kg) and caprylic acid (0.36 g/kg) combination in Queso fresco cheese [108]. Experiments with *L. innocua* showed that combinations of nisin with carvacrol, thymol, eugenol or cinnamic acids resulted in synergistic actions against the bacterium [123].

Carvacrol (30 mM), eugenol (32 mM), thymol (5 mM), thyrosol (0.5 mM) and terpineol (30 mM) paired with enterocin AS-48 (30 μg/g) reduced viable cell counts of *L. monocytogenes* CECT 4032 inoculated on Russian type salad at 10 °C by 3.57–4.57 log units compared to control after 24 h. In case of thymol, terpineol or thyrosol with enterocin, bacteria were no longer detectable. Reductions obtained in the combined treatments were significantly higher (*p* < 0.05) than the sum of reductions obtained for each treatment separately, indicating a more than additive effect [109].

### 5.3. Heat Treatments

There are several studies suggesting synergism between natural antimicrobials and mild heat treatments in inhibiting *L. monocytogenes*. Exponential growth of *L. monocytogenes* cells cultured at 8 °C showed reductions after 30 min mild heat (45 °C) treatment in the presence of antimicrobials, namely S-carvone, carvacrol, cinnamaldehyde, thymol or decanal, by 1.5 to 2.1 log units, even though reductions achieved by the separate treatments showed no effect [54]. Isothermal heating between 53 °C and 68 °C in the presence of citral, (E)-2-hexenal and carvarcrol at their sublethal concentrations reduced the time required to achieve 5-log reduction of *L. monocytogenes* counts, e.g., at 55 °C in the control the time was of 145.75 min, whereas in the presence of antimicrobials, the same pathogen reduction was achieved between 40.13 for (E)-2-hexenal and 42.11 min for citral [61]. Thymol or carvacrol (at 0.3 mM for each antimicrobial) combined with 55 °C treatment for 5, 10 and 15 min resulted in significantly higher percent reductions of the pathogen than with the mild heat treatment only. For example, 5 min treatment at 55 °C without additive, with thymol and with carvacrol resulted in 76.27%, 92.76% and 98.19% reduction of the pathogen, respectively [121].

Additionally, a Weibull model of thermal inactivation of *L. monocytogenes* ATCC 13932 in the presence of cinnamaldehyde in ground pork was created and showed that the supplementation of cinnamaldehyde in ground pork considerably increased the thermal susceptibility of *L. monocytogenes*. Four levels of temperature (55 °C to 70 °C) and four levels of cinnamaldehyde at concentrations of 0 up to 1% (vol/wt) were used to determine thermal inactivation curves of the pathogen. Time needed to achieve a 5-log pathogen reduction declined from 28.15 min to 17.35 min at the presence of 1% carvacrol compared to the sample without carvacrol. At 70 °C time required declined from 1.95 min to 0.27 min. Created secondary model, based on 5-log lethality was statistically significant (*p* < 0.0001) [88].

However, phenomena of cross-resistance caused by reaction to sublethal stress exists and due to this phenomena bacteria can gain cross-resistance to EOs or their components after sublethal heat treatment [124]. *L. monocytogenes* EGD-e was exposed to carvacrol at concentration of 200 μL/L at 4.0 or 7.0 pH buffer. Prior to the carvacrol exposure, bacteria were heat shocked at 45 °C for one hour. When cells were not heat shocked beforehand, they were reduced by more than 5 log CFU (to undetectable levels) at both pH values. Heat shocked cells however were reduced by approximately 2 log CFU at both pH [124].

### 5.4. Antibiotics

Natural plant-derived antimicrobials showed synergistic action with classic antibiotics. Oleanolic acid and ursolic acid used in combination with ampicillin and oxacillin increased susceptibility of *L. monocytogenes* PCM 2191 to these two antibiotics. Oleanolic acid or ursolic acid (both acids acted identically) at concentration of 1/2 MIC combined with antibiotics resulted in the decrease of the antibiotics MICs values by approximately 2-folds [125]. Additionally, gentamycin combined with erucin, sulforaphane and allylisothiocyanate showed increased antibacterial activity in relation to the action of the phytochemical alone [55]. Citral and carvacrol separately and in combination decreased erythromycin, bacitracin and colistin MIC values against *Listeria monocytogenes* CECT 4032. MIC values of 0.125, 32 and 96 μg/mL for erythromycin, bacitracin and colistin, respectively, were reduced to 0.002, 0.034 and 0.144, respectively, when 0.100 μg/mL carvacrol and 0.250 μg/mL were present in the culture medium, indicating approximately 62 to almost 1000 times increased sensitivity to antibiotics [126]. Streptomycin with thymol and cinnamaldehyde showed synergism, while eugenol and berberine with streptomycin showed additive effect. Synergy was sufficient to eradicate biofilm formed on polystyrene [53]. Citral at concentration of 0.250 μL/mL increased sensitivity of first and second generation of *L. monocytogenes* CECT 4032 to 8 antibiotics, out of 9 tested. MIC values of erythromycin, cephalothin, gentamicin, bacitracin, colistin, trimethoprim/sulfamethoxazole, ampicillin and ciprofloxacin were significantly reduced. However, sensitivity to chloramphenicol remained unchanged [127].

### 5.5. Other Factors

Other factors, such as high hydrostatic pressure (HHP), preservatives, condiments or bacteria in combination with NPDA also showed synergy. Combined treatments with carvacrol or thymol and HHP (150, 200, 250 or 300 MPa) at 1 °C, 8 °C or 20 °C on exponentially grown cells of *L. monocytogenes* were more effective than separate treatments. At 1 °C, more than 5 log reductions in viable counts were achieved by applying pressures of 250 MPa in combination with 2.5 or 3 mmol/L carvacrol or 300 MPa in combination with 2, 2.5 or 3 mmol/L carvacrol, whereas at higher temperatures achieved reductions were less pronounced. Additionally, combined treatments of HHP and thymol were most effective at 1 °C [92]. Combined treatment with carvacrol and HHP was also evaluated in milk [92] and ground chicken meat [98] as a model food, where synergism against *L. monocytogenes* was confirmed.

Soy sauce with thymol (1 mM) and soy sauce with carvacrol (1 mM), reduced bacteria counts to undetectable levels, i.e., by more than 7 log CFU/mL after 10 min of storage at 22 or 4 °C. Significant reduction have also been achieved by combination of soy sauce with eugenol at 4 °C, where bacteria were reduced by 2 log CFU/mL, whereas soy sauce alone and the compounds in saline alone were not effective in reducing *L. monocytogenes* [101].

Hydrogen peroxide at concentration of 0.1% resulted in much greater performance of β-resorcylic acid (1.5%), carvacrol (0.75%) and *trans*-cinnamaldehyde (0.75%) when used in combination as antilisterial agents in frankfurters, leading to complete eradication of the pathogen when dipping treatment was applied at 65 °C [102]. Similarly, hydrogen peroxide at 2% concentration used in combination with carvacrol, thymol, β-resorcylic acid, and caprylic acid in washing solution, resulted in eradication of the pathogen from cantaloupes rind, whereas these substances alone was less effective [110]. Phenolic compounds (thymol, carvacrol, and eugenol) also showed synergism with potassium sorbate against *Listeria innocua*. Experiments were performed in two levels of water activity (a_w_; 0.99 or 0.97) and two levels of pH (5.5 or 4.5). Potassium sorbate alone was inhibitory at 400–600 ppm, whereas phenolic compounds alone were inhibitory at concentrations of 200–250 ppm of eugenol, 100-200 ppm of thymol and 100–150 ppm of carvacrol, depending on the environmental conditions. At a_w_ of 0.99 and pH 5.5 *Listeria innocua* growth was inhibited by combination of 50 ppm of potassium sorbate combined with 50 ppm of phenolic compound (either thymol, carvacrol or eugenol), proving synergism between the compounds [128].

Carvacrol or gallic acid paired with chitosan also acted synergistically in *L. monocytogenes* inhibition, as MIC values of the two compounds used together were lower than it would result from an additive effect [103].

Lactic acid bacteria showed synergy with eugenol. Combination of eugenol at 0.04% with either of the lactic acid bacteria tested (*Bifidobacterim bifidum*, *Lactobacillus reuteri*, *Lactobacillus fermentum*, *Lactobacillus plantarum* or *Lactococcus lactis*) reduced invasion of Caco-2 cells to undetectable level, compared to approximately 2–3 log CFU/mL invasion rate when bacteria were used alone or 1.25 log CFU/mL when eugenol was used alone [93].

## 6. Alternative Delivery Methods of the Components Used against *L. monocytogenes*

Alternative ways of delivering antilisterial agents to the pathogen (compared to direct application of NPDA to food products or to laboratory medium) have been developed by many authors. Encapsulation and creating various films with plant derived compounds have been investigated and tested against *L. monocytogenes*. These systems are developed, because microorganisms proliferate in the aqueous phase of the food system and due to hydrophobic nature of most of the compounds, especially present in violate oils, mass transport of these active compounds to the microorganisms is relatively small [112]. Alternative ways of delivering are proposed to overcome this limitation, but also to prolong efficacies of the compounds, as they are hindered by their rapid depletion in foods [129]. Delivery systems also limit sensory impact of the antimicrobials on the products, as better delivery enable lower concentrations of antimicrobials.

Carvacrol and eugenol were solubilized in nonionic surfactant micelles (Surfynol 465 and 485W) and the activity of the encapsulated compounds depended on the surfactant used as a carrier. For carvacrol lower MICs were achieved with Surfynol 485W and varied from 0.025 to 0.35% (wt/wt), depending on the bacterial strain. For eugenol better results were achieved with Surfynol 465, where MICs varied from 0.1% to 0.3% [112]. Micellar-encapsulated carvacrol and thymol by nonionic surfactants [130,131], as well as carvacrol encapsulated into nanocapsules [80] have been also used as biofilm inhibitors. Citral and linalool alone or in combinations were nanoemulsified with Tween 80 and alternatively with ripening inhibitors, namely medium chain triglycerides, coconut oil, sesame oil or castor oil. Formulation containing citral only (stable throughout storage) showed the lowest MIC value out of formulation tested and reached 0.312%, whereas free citral showed MIC of 0.635%. Free linalool also showed greater MIC (of 1.25%) than emulsified (0.635%). MIC values of emulsions containing both antimicrobials was of 0.635%, whereas ripening inhibitors increased MIC value to 1.25%. Experiments were performed with *Listeria monocytogenes* ATCC 19111. The anti-biofilm activity of the free citral and citral nanoemulsion (citral at 5% concentration) indicated 81.54% and 83.51% biofilm inhibitions, respectively [132]. Carvacrol encapsulated into nanoliposomes (NLC) by the lipid film hydration technique and into nanocapsules (NCC) by the interfacial deposition technique of the preformed polymer had higher MBC values of 3.31 mg/mL for NCC and 5.30 mg/mL for NLC, compared to 1.77 mg/mL in the free carvacrol solution [80]. However, in the other study MIC value of polylactide nanoparticles with polyethylenimine coating and loaded with carvacrol was of 128 μg/mL, whereas free carvacrol had MIC of 1024 μg/mL against *L. monocytogenes*. Evaporation rates of the nanoparticles was less than 10% (weight) after 6 days at 37 °C, whereas free carvacrol was reduced to less than 25% [64]. Efficacy of nanoemultions of eugenol and thymol prepared with auric arginate and lecithin (which allowed to stabilize emulsion during storage) was evaluated in 2% reduced fat milk [106], as well as food grade capsules created by spray drying zein with coencapsulated nisin and thymol as antimicrobials, along with glycerin in some formulations were also evaluated in in milk [107] as described earlier in the text and presented in Table 5.

Poly(ethylene-co-vinylacetate) (EVA14) films, containing 3.5 wt% cinnamaldehyde or carvacrol and 7 wt% of carvacrol was not inhibitory against *L. monocytogenes*, however film containing 7 wt% of cinnamaldehyde resulted in antilisterial activity, with inhibition zone diameter of 15 mm in disc diffusion method. Authors also incubated the films with pathogens at 37 °C for up to 48 h, and relative inhibition of biofilm formed on the polymeric films compared to control was of minimum 48.4 for cinnamaldehyde at 3.5% after 24 h up to 76.3 for 7% carvacrol after 48 h [133]. Poly(lactic acid)-based nanofibres with incorporated 30% gelatin and 12% carvacrol, showed inhibitory effect against *L. monocytogenes* culture under the film, with average inhibitory zone of 35.9 mm^2^ [134].

Nanofibers of fish skin gelatin encapsulating carvacrol (15–30% *w*/*w*) were prepared by blow-spinning method. *L. monocytogenes* 19115 was inhibited by created nanofibers. Nanofiber mats were placed on agar plates and incubated at 35 °C for 24 h. Oppositely to most of the other reports, authors achieved the biggest inhibition zone when fibers had the least carvacrol concentration (15%) and the smallest with 30% of carvacrol [135]. However, the same group prepared also nanofibers containing cinnamaldehyde (5–30%) and measured activity of the fibers using the same method. In this case, inhibition zones were increasing along with increasing cinnamaldehyde concentration [136]. Poly(lactic) acid-based films containing cinnamaldehyde inclusions (CI) at a concentration of at least 10% CI concentration showed 100% inhibition of *L. monocytogenes* in vitro. What is more, CI at 10% significantly improved tensile strength of the film. Cinnamaldehyde release was the highest at the first day and then smoothly decreased thought 20 days of storage [111]. Carvacrol loaded on electrospun gelatinized soluble potato starch fibers had better thermal stability than the free compound. Growth reduction of *L. monocytogenes* reached 89.0% [137]. Pea protein isolate-polyvinyl alcohol nanofibers incorporating cinnamaldehyde inhibited *L. monocytogenes* in diffusion assay, as the diameter of the inhibition zones varied from 10 mm to 15 mm (for concentrations of 1 wt% to 1.5 wt% of carvacrol) [138]. An edible film of *Gelidium corneum* containing carvacrol was incorporated as ham package against *L. monocytogenes* [104], films made of apple, carrot and hibiscus purees, incorporated with carvacrol or cinnamaldehyde at 0.5%, 1.5%, and 3.0% concentrations were used to wrap cooked ham and bologna [105], but also cassava starch films containing chitosan along with of gallic acid, as well as films with chitosan and carvacrol were tested on cooked ham [103], as described earlier in the text and presented in Table 5.

## 7. Natural Substances against *L. monocytogenes* Biofilm

As already mentioned, *L. monocytogenes* is able to form biofilm, where bacteria anchor themselves to surfaces and each other and are covered in extracellular matrix, what makes them especially hard to remove [2,7]. Several experiments showed, that NDPA are able to reduce listerial biofilm and biofilm formation on stainless steel surfaces and on other materials.

On stainless steel, carvacrol at concentration 2 mM removed or inactivated 32% of *L. monocytogenes* dried biofilm [139], carvacrol- or eugenol-loaded micelles were shown to reduce mature *L. monocytogenes* films, whereas achieved reduction depended on contact time, bacterial strain and medium on which the biofilm was grown. Eugenol-loaded micelles after 20 min exposure time was able to reduce biofilm to undetectable levels [130]. On the other experiment however nanocapsules containing carvacrol resulted in initial reduction of the pathogen anchored to stainless steel, however within next 5 h of incubation the counts were similar to control untreated sample. At the end of the experiment, after 25 h, the two samples showed no differences, reaching approximately 10 log CFU/cm^2^ [80]. Sub-inhibitory concentrations of *trans*-cinnamaldehyde, carvacrol, thymol and eugenol showed inhibitory effect during biofilm formation on stainless steel and the inhibitory effect was more pronounced at 4 °C growth temperature (compared to 25 and 37 °C). Against pre-formed biofilm, carvacrol, thymol and eugenol resulted in pathogen reduction to undetectable levels after 15 min of incubation at all three temperatures. *Trans*-cinnamaldehyde resulted in reduction of *L. monocytogenes* counts, however in all cases the pathogen was still detectable at all temperature levels [79]. Combined action of cinnamaldehyde and eugenol resulted in approximately 90% inhibition of *L. monocytogenes* on the surface of 96-well microtiter plate [66].

The adherence ability of *L. monocytogenes* was also significantly (*p* < 0.05) inhibited by eugenol and citral at the subinhibitory concentration (0.05 mg/mL). Strong reduction (*p* < 0.05) of the adherence was observer after exposure to the MBC concentration of eugenol or citral during 30 min [68]. Resveratrol at its MIC value of 200 mg/L inhibited approximately 75% of biofilm formed in wells of flat-bottom 96-well polystyrene microtiter plate. Relative biofilm cell metabolic activity of treated strains was reduced to approximately 30% compared to the untreated control [74]. *Trans*-cinnamaldehyde, terpinene-4-ol or thymol, at their sub-inhibitory concentrations resulted in significant biofilm reduction compared to the control, when they were added to suspensions of 24 h-old *L. monocytogenes* culture. *Trans*-cinnamaldehyde performed the best, then followed by (not significantly different between each other) terpinene-4-ol and thymol [67]. Additionally, light controllable micelles incorporating thymol were tested against *L. monocytogenes* biofilm. Micelles were made of chitosan, toluidine blue O and poly(propylene sulfide) and activated under irradiation, which generates reactive oxygen species (ROS), which result in additional ROS-related bactericidal effects and stimulate thymol release. *L. monocytogenes* biofilm treated with these micelles after irradiation at 20 mW/cm^2^ for 10 min was reduced to approximately 0.3 relative biofilm value compared to the control untreated with micelles, but also irradiated [140].

Experiments focusing on inhibiting *L. monocytogenes* biofilm are usually based on created homogenous mono-species systems. However, in vivo biofilms are heterogeneous, comprising different microorganisms forming a complex multi-species community [67]. Kerekes et al. [67] created dual-species biofilms where *L. monocytogenes* was paired with *Escherichia coli*, *Pseudomonas putida* and *Staphylococcus aureus*. Activity of *trans*-cinnamaldehyde, terpinene-4-ol and thymol were tested against these biofilms. Inhibition of biofilm formed with *E. coli* (i.e., eradication of both microorganisms) was achieved by cinnamaldehyde at concentration 0.5 mg/mL, terpinen-4-ol at 8 mg/mL and thymol at 0.2 mg/mL. Biofilm with *S. aureus* was inactivated by cinnamaldehyde at concentration of 0.8 mg/mL, terpinen-4-ol at 0.5 mg/mL and thymol at 8 mg/mL. Biofilm with *P. putida* was inactivated by terpinen-4-ol at 0.1 mg/mL and thymol at 8 mg/mL. Cinnamaldehyde at concentration of 1 mg/mL completely inhibited *L. monocytogenes*, however *P. putida* in the biofilm still survived at concentration of ca. 5.5 log CFU/mL.

## 8. Summary and Conclusions

Many natural chemical compounds show inhibitory or bactericidal activity against *L. monocytogenes* and bacterial cells are highly influenced by the presence of the compounds in the environment. Primary mechanism of action of eugenol, carvacrol [86], cinnamaldehyde [88], citral and (E)-2-hevenal [89] is cell membrane disruption.

Some of the components influence fatty acid composition of cell membranes [90,91], change gene expression, including down-regulation of virulence genes [58,59,77,79,93], change bacterial proteome [94] and change levels of biogenic amines production [95]. Presence of the antimicrobials also reduce adhesion and invasion of human cell cultures [59,77,93].

The antimicrobials were used in many food model systems and in some cases complete eradication of the pathogen was achieved, even though food components inhibit action of the antimicrobials [52,63,70,74,92,112]. *L. monocytogenes* at the end of storage was no longer detectable after treatment with NPDA in raw meat [98,99], teriyaki sauce [99] and in soy sauce [101], in raw fish [100], various meat products [102,105,111], milk [70], salads and vegetable models [65,69,109] and cantaloupes [110]. However, there are no studies where the effect of NPDA at undetectable (in terms of sensory analysis) levels would be measured in food products contaminated with *L. monocytogenes* and such experiments would be valuable.

Achieving sufficient reductions of the pathogen in food model systems without any sensory impact would probably require using binary or ternary combinations of the natural antimicrobials, as many of them show synergism in combination [41,52,65,76,114,119,120,121] or combining them with bacteriocins, as bacteriocins also act synergistically with many NPDA [23,41,57,70,75,108,109,122,123]. Other factors, for example mild heat treatments also significantly enhance action of the NPDAs [61,88,92,121]. Alternative methods of delivering the compounds, e.g., microcapsulation, may also reduce sensory impact of the antimicrobials on the food product, as they increase mass transport of active compounds to the microorganisms and due to that concentration of the antimicrobial can be decreased [112].

NPDA also reduce mature *L. monocytogenes* biofilm or inhibit biofilm formation [2,7,66,67,68,74,80,130,139,140] and thus could potentially be used during washing processes at food processing facilities.

Even though many publications suggest that NPDA could be very beneficial in combating *L. monocytogenes*, there are also two disturbing finding. Firstly, *C. elegans* showed loss in life span (*p* < 0.05) when it was fed in a lawn of *L. monocytogenes* previously treated carvacrol compared to untreated cells. Additionally, carvacrol treated bacteria reduced statistically significantly the number of laid eggs [97]. Second disturbing finding is that in presence of *trans*-cinnamaldehyde at concentrations close to MBC, *L. innocua* cells entered in the VBNC state [87]. This finding raises a question if *L. monocytogenes*, closely related to *L. innocua*, in other studies could also survive but was not enumerated, because of methodology limitations. Though some studies indicate that VBNC *L. monocytogenes* are avirulent [141,142] other study showed that ingestion of VBNC *L. monocytogenes* by *C. elegans* resulted in significant reduction in life span, that was not different from life span reductions caused by culturable *L. monocytogenes* treatments [143].

Areas that require further studies in the field of NPDA as *L. monocytogenes* inhibitors are especially: ability of the pathogen to enter VBNC state in food products treated with NPDA and also studies focused on creating NPDA food treatments that would be both completely undetectable by consumers and effective against the pathogen.

## Figures and Tables

**Table 1:** Example plant sources of chosen antilisterial plant-derived compounds.

Plant Source (Latin Name)	Plant Source (Common Name)	Compound	Concentration in the EO (%)	References
*Thymus vulgaris, Origanum vulgare*	thyme, oregano	carvacrol	2–11 trace–80	[13,22]
*Anethum graveolens* L.	dill seed	carvone S	45.5	[22,46]
*Cinnamomum zeylandicum*	cinnamon	cinnamaldehyde *trans*-cinnamaldehyde	65	[13,22]
*Cymbopogon citratus*	lemongrass	citral	65–85	[22,47]
*Eugenia* *caryophyllata* (*Syzigium aromaticum* L. Myrtaceae)	clove	eugenol	88.6	[48]
*Lavandula angustifolia*	lavender	linalool	5–57	[49]
*Thymus vulgaris, Origanum vulgare*	thyme, oregano	thymol	10–64, trace–64	[22]
*Psidium guajava* L.	guava fruits	(E)-2-hexenal	7.4	[50]

**Table 2:** Values of BA50 of plant-derived compounds against *L. monocytogenes* ^1^.

Compound	BA50 Approximate Range (μg/mL) ^2^	BA50 as Defined in References (%) ^3^
thymol	70	0.007
cinnamaldehyde	80–190	0.008–0.019
4-hydroxytyrosol ^4^	260	0.026
eugenol	610–810	0.061–0.081
carvacrol	830–860	0.083–0.086
citral	990–2000	0.099–0.20
carvone S	1700–3500	0.17–0.35
limonene	2500 (–>6700)	0.25 (–>0.67)
geraniol	2800–5100	0.28–0.51
perillaldehyde	3000–3500	0.30–0.35
estragole	3500–3600	0.35–0.36
benzaldehyde	3600–4600	0.36–0.46
salicylaldehyde	4300–4500	0.43–0.45
cironella S	4400–7000	0.44–0.70
cironella R	4500–12,000	0.45–1.2
menthol	4800–5700	0.48–0.57

^1^ Where not mentioned otherwise, the experiments were performed with two bacterial strains, namely: RM2199 (F2379), RM2388 and information was taken from Reference [22]; ^2^ Values were converted for ease of comparison with Table 3 and Table 4 within the paper; density was assumed as 1 g/mL for all of the compounds; ^3^ Presented range of the values refers to results obtained for different strains used in the study; ^4^ the experiment were performed with two strain RM2199 and the information was taken from Reference [40].

**Table 3:** Minimal Inhibitory Concentrations of plant-derived compounds against *L. monocytogenes* strains.

Compound	MIC Approximate Range ^1^ (μg/mL)	MIC as Defined in References ^2^	Strains(s) Used in the Reference Study	References
carvacrol	65	65 μg/mL	ATCC 15313	[57]
	100	100 mg/L	Scott A	[58]
	113	0.75 mM	ATCC 19115, Scott A, Presque-598	[59]
	125	0.125 μL/mL	NCTC 11994, S0580	[60]
	125–300	125–300 mg/L ^3^	56LY	[61]
	156–625	156–625 μg/mL	EGDe, LO28, F2356, 33413, 33013	[62]
	245	1.63 mM	Isolate was not defined ^4^	[63]
	256–>1024	256–>1024 μg/mL ^5^	ATCC 7644	[64]
	600	0.6 μL/mL	ATCC 7644	[65]
cinnamaldehyde	65	65 μg/mL	ATCC 15313	[57]
	83	83.33 μg/mL	MTCC 657	[66]
	500–1000	500–1000 ppm	ATCC 15313, H7962, NADC 2045 (Scott A)	[41]
	512	512 μg/mL	CMCC 54004	[53]
*trans*-cinnamaldehyde	119	0.90 mM	ATCC 19115, Scott A, Presque-598	[59]
	125–250	0.125–0.25 μL/mL	NCTC 11994, S0580	[60]
	156–312	156–312 μg/mL	EGDe, LO28, F2356, 33413, 33013	[62]
	250	0.25 mg/mL	SZMC 21307	[67]
citral	60–80 80–100	0.06–0.08 mg/mL 0.08–0.1 mg/mL ^6^	EGD, Scott A, C882	[68]
	225–400	225–400 mg/L ^3^	56LY	[61]
	250	250 mg/L	Scott A	[58]
	300	0.03% *v*/*v*	ATCC 7644 (C3970)	[69]
eugenol	67	66.66 μg/mL	MTCC 657	[66]
	80	0.08 mg/mL	EGD, Scott A, C882	[68]
	90	90 μg/mL	ATCC 15313	[57]
	500	0.5 μL/mL	NCTC 11994, S0580	[60]
	1024	1024 μg/mL	CMCC 54004	[53]
linalool	750–1000	0.75–1 μL/mL	NCTC 11994, S0580	[60]
	2500	0.25% *v*/*v*	ATCC 7644 (C3970)	[69]
thymol	90	0.60 mM	ATCC 19115, Scott A, Presque-598	[59]
	150	150 μg/mL	ATCC 15313	[57]
	156–312	156–312 μg/mL	EGDe, LO28, F2356, 33413, 33013	[62]
	250	0.25 mg/mL	Scott A	[70]
	250–800	250–800 μg/mL	ATCC 7644, ATCC 19114, ATCC 19115, NCTC 10887, NCTC 18890 and 25 strains from different foods ^7^	[71]
	250	0.25 μL/mL	NCTC 11994, S0580	[60]
	500	0.5 mg/mL	SZMC 21307	[72]
	1024	1024 μg/mL	CMCC 54004	[53]
(E)-2-hexenal	325–1400	325–1400 mg/L ^3^	56LY	[61]
	800	800 mg/L	Scott A	[58]
1,8-cineole	20,000	20 μL/mL	ATCC 7644	[65]
β-caryophyllene	167	166.66 μg/mL	MTCC 657	[66]
3β-acetylursolic acid	1670	1.67 mg/mL	ATCC 19115	[73]
methyl-3β-hydroxylanosta-9,24-dienoate	185	0.185 mg/mL	ATCC 19115	[73]
3β-hydroxylanosta-9,24-dien-21-oic acid	185	0.185 mg/mL	ATCC 19115	[73]
resveratrol	200	200 mg/L	LMG 16779, LMG 16780, LMG 13305	[74]
diglicerol monolaurate	50	0.005%	IID 581	[75]
monolaurin	31–125	31.25–125 μg/mL ^8^	ATCC 19118	[76]
terpinene-4-ol	4000	4 mg/mL	SZMC 21307	[67]
thymoquinone	6–13	6.25–12.50 μg/mL	ATCC 19115, ATCC 15313 and 6 strains from different foods ^9^	[77]
berberine	8192	8192 μg/mL	CMCC 54004	[53]

^1^ Values were converted for ease of comparison; density was assumed as 1 g/mL for all of the compounds, and molecular weights were obtained from PubChem [78]; ^2^ Presented range of the values, if not specified otherwise, refers to results obtained for different strains used in the study; ^3^ Depending on the level of the initial contamination—from 2 to 6 log CFU/mL (higher contamination resulted in higher MICs); ^4^ Authors described the isolate as “Dutch field isolate obtained from cheese”; ^5^ Depending on the time of the experiment (0–72 h)—longer inhibition required higher doses; ^6^ Lower range was obtained by microdilution method, higher by agar dilution method; ^7^ 25 strains of food origin (from meat, vegetables, dairy and fish) were described as: 45, 46, 47, 48, 49, 50, 51, 52, 53, 79, 85, 89, 96, 98, 99, 100, 101, 102, 103, 104, 105, 106, 107, 112 and 118 in the reference study; ^8^ MIC was determined at 2 temperatures (5 °C and 30 °C) and 3 levels of pH (5.0, 6.0 and 7.0); the lowest MIC was achieved at 5 °C at pH 5, whereas the highest at 30 °C at pH 7; ^9^ 5 strains of food origin (from raw chicken meat, infant food, and ready-to-eat meals) were marked as: A17, A24, B9, B19, C6, and C34.

**Table 4:** Minimal Bactericidal Concentrations of plant-derived compounds against *L. monocytogenes* strains.

Compound	MBC, Approximate Range ^1^ (μg/mL)	MBC as Defined in References ^2^	Strains(s) Used in the Study	References
carvacrol	150–300	150–300 mg/L ^3^	56LY	[61]
	250	0.25 μL/mL	NCTC 11994, S0580	[60]
	255	1.7 mmol/L	IID 581	[75]
	751	5.0 mM	ATCC 19115, Scott A, Presque-598	[79]
	1770	1.77 mg/mL	MBC was determined for bacterial pool of strains: ATCC 7644, 7459 and J11	[80]
cinnamaldehyde	92	91.66 μg/mL	MTCC 657	[66]
	1004	7.6 mmol/L	IID 581	[75]
	3965	30 mM	CRIFS C717	[81]
*trans*-cinnamaldehyde	500	0.5 μL/mL	NCTC 11994, S0580	[60]
	661	5.0 mM	ATCC 19115, Scott A, Presque-598	[79]
citral	80–100	0.08–0.1 mg/mL	EGD, Scott A, C882	[68]
	250–450	250–450 mg/L ^3^	56LY	[61]
eugenol	67	66.66 μg/mL	MTCC 657	[66]
	100	0.1 mg/mL	EGD, Scott A, C882	[68]
	821	5 mM	CRIFS C717	[81]
	1000	1 μL/mL	NCTC 11994, S0580	[60]
	1002	6.1 mmol/L	IID 581	[75]
	3087	18.5 mM	ATCC 19115, Scott A, Presque-598	[79]
isoeugenol	1248	7.6 mmol/L	IID 581	[75]
thymol	496	3.3 mmol/L	IID 581	[75]
	496	3.3 mM	ATCC 19115, Scott A, Presque-598	[79]
	500	0.5 μL/mL	NCTC 11994, S0580	[60]
	500	0.5 mg/mL	Scott A	[70]
(E)-2-hexenal	325–1400	325–1400 mg/L ^3^	56LY	[61]
diglycerol monocaprinate	200	0.02%	IID 581	[75]
diglicerol monolaurate	100	0.01%	IID 581	[75]
diglycerol monomyristate	200	0.02%	IID 581	[75]

^1^ Values were converted for ease of comparison; density was assumed as 1 g/mL for all of the compounds, and molecular weights were obtained from PubChem [78]; ^2^ Presented range of the values, if not specified otherwise, refers to results obtained for different strains used in the study; ^3^ Depending on the level of the initial contamination—from 2 to 6 log CFU/mL (higher contamination resulted in higher MICs).

**Table 5:** Summarized attempts of inhibiting *Listeria monocytogenes* in food model systems.

Row	Food Category	Food Product Description	Approx. Initial Contamination ^1^	Storage Time and Temp.	Treatment(s) That Resulted in the Most Significant Pathogen Reduction ^2^	Approx. Final Contamination ^1^		Ref.
						In Food Product	In Control Sample	
AA	Meat	Fresh ground chicken meat (ca. 98% lean, 2% fat, without additives)	10^7^ to 10^8^ CFU/g	3 days 10 °C	0.75% (*w*/*w*) carvacrol	6.27 log CFU/g	7.94 log CFU/g	[98]
AB	Meat	Fresh ground chicken meat (ca. 98% lean, 2% fat, without additives)	10^7^ to 10^8^ CFU/g	7 days 4 °C	0.75% (*w*/*w*) carvacrol + 300 MPa OR 0.60% or 0.75% (*w*/*w*) carvacrol + 350 MPa	n.d. ^3^ (<1 log CFU/g)	7 log CFU/g (in S ^4^ treated with HPP only)	[98]
AC	Meat	Fresh ground chicken meat (ca. 98% lean, 2% fat, without additives)	10^7^ to 10^8^ CFU/g	7 days 10 °C	0.60% or 0.75% (*w*/*w*) carvacrol + 350 MPa	n.d. (<1 log CFU/g)	8 log CFU/g (in S treated with HPP only)	[98]
AD	Meat	Fresh, skinless chicken breast fillets	10^2^ CFU/cm^2^ before 24 h bacterial pregrowth	48 h 6 °C	0.25 mg/mL thymol + 5% (*w*/*v*) salt ^5^	2.2 log CFU/cm^2^ (non-washed S); 3.0 log CFU/cm^2^ (washed S)	4.9 log CFU/cm^2^ (non-washed S); 3.9 log CFU/cm^2^ (washed S)	[72]
AE	Meat	Beef sirloins	3.3 log CFU/g	7 d 4 °C	0.5% thymol ^5^	n.d.	3.4 log CFU/g (not marinated S); 2.7 log CFU/g (S marinated in teriyaki sauce)	[99]
AF	Meat	Raw pork loin	10^5^ CFU/g	12 h 4 °C	15 ppm carvacrol + 6 ppm grapefruit seed extract + 4.5 ppm nisin ^5^	2.88 log reduction achieved	N/A ^6^	[41]
AG	Meat	Steak tartare (ground beef mixed with Filet Américain sauce)	10^7^ CFU/g	4 weeks 10 °C	5 mmole/g carvacrol	10^8^ CFU/g	10^8^ CFU/g	[63]
AH	Skin	Heat sterilized chicken skin	8.0 log CFU/sample	N/A	0.25% (*v*/*v*) linalool ^5^	6.2 log CFU/sample	not presented	[69]
AI	Fish	Channel catfish fillets	3.9 log CFU/g	10 d 4 °C	2% carvacrol ^5^	n.d. (<1.3 log CFU/mL)	5.5 log CFU/g	[100]
AJ	Marinating solution	Marinade containing 5% (*w*/*v*) salt and thymol; control sample contained salt only	N/A (3.3 log CFU/cm^2^ in control Ss after 24 h)	48 h 6 °C	0.25 mg/mL thymol + 5% (*w*/*v*) salt ^5^	2.1 log CFU/cm^2^	4.0–5.0 log CFU/cm^2^	[72]
AK	Meat juice	Centrifuged and filtered chicken juice from defrosted commercially frozen chickens without viscera	6.5 log CFU/mL	14 d 4 °C	400 mg/L resveratrol OR 200 mg/L resveratrol	6 log CFU/mL	8.5 log CFU/mL	[74]
AL.	Soy sauce	Commercially available preservative-free soy sauce	7.3 log CFU/mL	14 d 22 °C or 4 °C	1 mM carvacrol OR 1 mM thymol	n.d.	0 log CFU/mL reduction achieved	[101]
AM	Teriyaki sauce	Commercially available sauce (soy sauce, wine, high fructose corn syrup, water, vinegar, salt, spices, onion powder, and garlic powder)	0 log CFU/mL	7 d 4 °C	0.3% or 0.5% carvacrol OR 0.3% or 0.5% thymol	n.d.	3.1 log CFU/mL	[99]
AN	Meat product	Fresh, skinless, pork–beef frankfurters (20% fat)	6 log CFU/frankfurter	70 d 4 °C	0.75% trans-cinnamaldehyde + 0.1% hydrogen peroxide ^5^	n.d.	7.3 log CFU/frankfurter	[102]
AO	Meat product	Fresh, skinless, pork–beef frankfurters (20% fat)	6 log CFU/frankfurter	70 d 4 °C	0.75% trans-cinnamaldehyde + 0.1% hydrogen peroxide OR 0.75% carvacrol + 0.1% hydrogen peroxide ^5^	n.d.	6 log CFU/frankfurter	[102]
AP	Meat product	Cooked ham with 3% (*w*/*w*) sodium chloride	2.5 log CFU/cm^2^	28 d 4 °C	0.195 g carvacrol + 0.025 g chitosan OR 0.1 g gallic acid+ 0.15 g chitosan (amounts of per 1 g of starch in film)	n.d. (<100 CFU/cm^2^)	9 log CFU/cm^2^	[103]
AQ	Meat product	Ham	7.3 log CFU/g	9 d 4 °C	0.6% carvacrol (concentration in film)	4.5 log CFU/g	6.1 log CFU/g	[104]
AR	Meat product	Cooked ham	5.0 log CFU/g	7 d 4 °C	3% carvacrol (in apple or hibiscus or carrot film)	n.d. (<1.3 log CFU/g)	4.4–4.6 log CFU/g (depending on the type of film)	[105]
AS	Meat product	Bologna	5.0 log CFU/g	7 d 4 °C	3% carvacrol in hibiscus film OR 3% carvacrol in apple film (OR 1.5% carvacrol in hibiscus film)	2.25 log CFU/g (3.0 log CFU/g)	4.5–4.8 log CFU/g (depending on the type of film)	[105]
AT	Milk	Pasteurized cow milk type A; 3% fat	6 log CFU/mL	6 d 4 °C	37.5 μg/mL thymol + 31.25 μg/mL nisin	4 log CFU/mL	6 log CFU/mL	[57]
AU	Milk	Ultra-high-temperature (UHT) processed 2% reduced-fat milk	6.5 log CFU/mL	120 h 21 °C	2 mg/mL thymol + 500 IU/mL nisin + 10 mg/mL lactobionic acid OR 2 mg/mL thymol + 250 IU/mL nisin + 10 mg/mL lactobionic acid	n.d. (<1 log CFU/mL)	8.75 CFU/mL	[70]
AV	Milk	Ultra-high-temperature (UHT) whole milk	6.5 log CFU/mL	120 h 21 °C	2 mg/mL thymol + 500 IU/mL nisin + 10 mg/mL lactobionic acid	2 log CFU/mL	8.3 CFU/mL	[70]
AW	Milk	Ultra-high-temperature (UHT)-treated skim milk	6.2 log CFU/mL	14 d 4 °C	400 mg/L resveratrol	7.8 log CFU/mL	8.5 log CFU/mL	[74]
AX	Milk	Ultra-high-temperature (UHT)-treated whole milk	6.2 log CFU/mL	14 d 4 °C	400 mg/L resveratrol OR 200 mg/L resveratrol	8.0 log CFU/mL	8.3 log CFU/mL	[74]
AY	Milk	Semi-skimmed milk	not presented	N/A	3 mmol/L carvacrol + 300 MPa HHP	3.2 log reduction in viable counts achieved	0.0 log reduction in viable counts achieved	[92]
AZ	Milk	2% reduced fat milk	5.5 log CFU/mL	120 h 21 °C	750 ppm of nanoemultions containing 1% of eugenol or thymol and other compounds (see comments in Table 6)	1.2 log CFU/mL	8.8 log CFU/mL	[106]
BA	Milk	2% reduced fat UHT milk	6.3 log CFU/mL	48 h 25 °C	capsules prepared by spray drying nisin extract with 1% *w*/*v* thymol (and other compounds—see comments in Table 6); concentration of capsules was adjusted to nisin concentration of 400 IU/mL	8.1–8.5 log CFU/mL	9 log CFU/mL	[107]
BB	Cheese	Queso fresco cheese manufactured by authors	3.2 log CFU/g–4.3 log CFU/g (depending on bacterial cocktail)	20 days 4 °C	0.72 g/kg caprylic acid + 0.49 g/kg nisin	2.2 log CFU/g–4.4 log CFU/g (depending on bacterial cocktail)	8.6 log CFU/g–8.2 log CFU/g (depending on bacterial cocktail)	[108]
BC	Cheese	Queso fresco cheese manufactured by authors	3.8 log CFU/g	20 days 4 °C	0.6 g/kg trans-cinnamaldehyde + 0.36 g/kg caprylic acid + 0.49 g/kg nisin	3.6 log CFU/g	7.0 log CFU/g	[108]
BD	Salad	RTE Russian salad of pH 4.44 (boiled: potatoes, carrots, peas, egg and raw olives, with mayonnaise)	4.67 log CFU/g	24 h 10 °C	5 mM thymol + 20 μg/g enterocin AS-48 OR 30 mM terpineol + 20 μg/g enterocin AS-48 OR 0.5 mM tyrosol + 20 μg/g enterocin AS-48	n.d.	4.57 log CFU/g	[109]
BE	Leafy vegetables	Fresh mix of iceberg lettuce, chard and rocket (in a rate of 1:1:1), hand shredded	not presented	N/A	0.6 μg/mL carvacrol ^5^	n.d. (<2 log CFU/g)	8.5 log CFU/g	[65]
BF	Vegetable model	Mix of iceberg lettuce, chard and rocket (60 g of each) mixed with 400 mL of water, blended, filtered and sterilized by filtration	6.2 log CFU/mL	24 h 37 °C	0.6 μg/mL carvacrol OR 20 μg/mL 1,8-cinole OR 0.075 μg/mL carvacrol + 2.5 μg/mL 1,8-cinole OR 0.15 μg/mL carvacrol + 5 μg/mL 1,8-cinole	n.d. (<2 log CFU/g)	8.2 log CFU/g	[65]
BG	Vegetable model	Iceberg lettuce (50 g) homogenized with 100 mL of water, adjusted to pH 7.2 by phosphate buffer, then autoclaved	6.2 log CFU/mL	14 d 4 °C	400 mg/L resveratrol OR 200 mg/L resveratrol	6.2 log CFU/mL	7.2 log CFU/mL	[74]
BH	Vegetables	Cabbage leaves (Sweetheart)	8.0 log CFU/sample	N/A	0.25% (*v*/*v*) linalool ^5^	n.d.	not presented	[69]
BI	Vegetables	Cabbage leaves (Sweetheart)	8.0 log CFU/sample	24 h 37 °C	1.85 mL/L beaker of citral OR 1.85 mL/L beaker of linalool	2.2 log CFU/sample	not presented	[69]
BJ	Vegetable	Lettuce leaves	10^5^ CFU/g	12 h 4 °C	15 ppm carvacrol + 6 ppm grapefruit seed extract + 4.5 ppm nisin ^5^	5 log reduction achieved	N/A	[41]
BK	Fruit	Red delicious apples, sliced	565 CFU/mL	10 days 5 °C	500 μg/mL rosmaric acid ^5^	28 CFU/mL	uncountable	[56]
BL	Fruit	Cantaloupe rind plugs prepared from fresh, whole cantaloupes,	7.3 log CFU/cm^2^	N/A	2% caprylic acid (alone or + 2% hydrogen peroxide) OR 2% thymol (alone or + 2% hydrogen peroxide) OR 2% carvacrol + 2% hydrogen peroxide ^5^	n.d.	4.9 log CFU/cm^2^	[110]
BM	Fruit	Cantaloupe rind plugs prepared from fresh, whole cantaloupes,	7.1 log CFU/cm^2^	7 d 4 °C	2% caprylic acid (alone or +2% hydrogen peroxide) OR 2% thymol (alone or +2% hydrogen peroxide) OR 2% carvacrol (+2% hydrogen peroxide) ^5^	n.d.	7.3 log CFU/cm^2^	[110]
BN	Juice	Steam-heated filtered carrot juice from fresh, raw Nantesa carrots, that were peeled, washed and minced	N/A (8.2 log CFU/mL in control sample after 24 h)	24 h 30 °C	1.6 mmol/L carvacrol + 1.6 mmol/L cymene	2.5 log CFU/mL	8.2 log CFU/mL	[52]

^1^ contamination refers to *L. monocytogenes* bacteria; ^2^ the column refers to the treatment or treatments that were the most effective at the end of the experiment; ^3^ n.d.—bacteria were not detected; ^4^ S—sample; ^5^ concentration refers to dipping or coating solution or sauce in which the food product was immersed; ^6^ N/A—not applied.

**Table 6:** Table with information supplementary to Table 5.

Row	*Listeria monocytogenes* Strain(s) Used in the Study	Other Treatments Presented in the Study ^1^	Comments	Ref.
AA	4-strain cocktail of: F4243, F4249, ATCC 7644, ATCC 43256	0.15%, 0.30%, 0.45% and 0.60% carvacrol	Chicken meat was manually mixed with targeted amount of carvacrol	[98]
AB	4-strain cocktail of: F4243, F4249, ATCC 7644, ATCC 43256	Combinations of 0.15%, 0.30%, 0.45% or 0.60% carvacrol +300 MPa or 350 MPa	Chicken meat was manually mixed with targeted amount of carvacrol	[98]
AC	4-strain cocktail of: F4243, F4249, ATCC 7644, ATCC 43256	Combinations of 0.15%, 0.30%, 0.45% or 0.60% carvacrol +300 MPa or 350 MPa	Chicken meat was manually mixed with targeted amount of carvacrol	[98]
AD	NCAIM B01934	N/A ^2^	Samples were initially washed with water (or not), inoculated and kept for 24 h at 6 °C, and then marinated in marinates containing 5% (*w*/*v*) salt and thymol; control sample contained salt only	[72]
AE	3-strain cocktail of: ATCC 19111, 19115, 19117	0.3% thymol 0.3% and 0.5% carvacrol ^3^	Samples were inoculated, kept overnight at 4 °C and then marinated (or not) in marinates containing teriyaki sauce alone or with carvacrol or thymol	[99]
AF	3- strain cocktail of: ATCC 15313, H7962, NADC 2045 (Scott A)	Various combinations of carvacrol (1.6–18 ppm) + grapefruit seed extract (0.64–7.36) + nisin (1.6–18.4) ^2^	90 mL of an antibacterial solution was added to 10 g samples	[41]
AG	Isolate was not defined, (described as “Dutch field isolate obtained from cheese”)	N/A	Control samples and treated samples were equally contaminated at the end of storage	[63]
AH	ATCC 7644 (C3970)	0.03% (*v*/*v*) citral ^3^	Effects of 60 s subjecting to antimicrobial solutions were measured; samples were not stored after treatment	[69]
AI	4-strain cocktail of not defined strains (described as serotypes 1/2b, 3b, 4b, and 4c previously “isolated from catfish processing facilities”)	1% carvacrol ^3^	Inoculated samples were dipped in 30 mL of carvacrol solution for 30 min at 4 °C; after treatment the solutions were drained out	[100]
AJ	NCAIM B01934	N/A	Inoculated chicken samples (washed or not prior inoculation) were immersed in the solution	[72]
AK	LMG 16779 serovar 1/2a	N/A	-	[74]
AL	3-strain cocktail of: ATCC 19111, 19115, 19117	1 mM eugenol; 1 mM trans-cinnamaldehyde; 1 mM β-resorcylic acid; 1 mM of vanillin	Substances other than carvacrol and thymol were not able to effectively reduce the pathogen during 10 min treatment and were not tested up to 14 days	[101]
AM	3-strain cocktail of: ATCC 19111, 19115, 19117	N/A	Inoculated beef was marinated in the sauce and leftover marinade was stored and examined	[99]
AN	5-strain cocktail of: ATCC Scott A, ATCC 19115, 101, 1, Presque-598	0.75% trans-cinnamaldehyde; 0.75% carvacrol; 0.75% carvacrol + 0.1% hydrogen peroxide ^3^	Frankfurters were immersed in dipping solutions for 60 s and held at 55 °C	[102]
AO	5-strain cocktail of ATCC Scott A, ATCC 19115, 101, 1, Presque-598	0.75% trans-cinnamaldehyde; 0.75% carvacrol ^3^	Frankfurters were immersed in dipping solutions for 30 s and held at 65 °C	[102]
AP	5-strain cocktail of: FSL J1-177, FSL C1-056, FSL N3-013, FSL R2-499, FSL N1-227	or 0.3 g gallic acid 0.3 g gallic acid + 0.025 g chitosan 0.048 g carvacrol + 0.025 g chitosan (amounts of antimicrobial per 1 g of starch in film)	Ham was covered with film made of cull potato starch	[103]
AQ	KCTC 3710	0.4% carvacrol (concentration in film)	Ham was covered with film made of *Gelidium corneum*	[104]
AR	strain 101M; serotype 4b	0.5%, 1.5% and 3% cinnamaldehyde 0.5% and 1.5% carvacrol (concentration in films)	Ham was covered with apple, carrot, or hibiscus films incorporated with carvacrol or cinnamaldehyde	[105]
AS	strain 101M; serotype 4b	Other combinations of 0.5%, 1.5% or 3% either cinnamaldehyde or carvacrol in all types of films	Bologna was covered with apple, carrot or hibiscus films incorporated with carvacrol or cinnamaldehyde;	[105]
AT	ATCC 15313	(37.5 μg/mL thymol or 16.25 μg/mL carvacrol or 22.5 μg/mL eugenol or 16.25 μg/mL cinnamaldehyde) + 31.25 μg/mL nisin	-	[57]
AU	Scott A	Nisin (up to 500 IU/mL), thymol (up to 10 mg/mL) and lactobionic acid (up to 10 mg/mL) alone or in binary and ternary combinations	-	[70]
AV	Scott A	Nisin (up to 500 IU/mL), thymol (up to 10 mg/mL) and lactobionic acid (up to 10 mg/mL) alone or in binary and ternary combinations	-	[70]
AW	LMG 16779 serovar 1/2a	200 mg/L resveratrol	-	[74]
AX	LMG 16779 serovar 1/2a	N/A	Differences in final bacterial counts in treated and control samples were not statistically significant	[74]
AY	Scott A	3 mmol/L carvacrol; 300 MPa HHP	Effects of 20 min treatment with antimicrobial and HHP (separately or in combination) at 1 °C were measured; samples were not stored after treatment	[92]
AZ	Scott A	N/A	Nanoemultions were prepared with 1% *w*/*w* of thymol or eugenol and 0.93% *w*/*w* lauric arginate and 1% *w*/*w* lecithin	[106]
BA	Scott A	N/A	Treatment was done with food-grade capsules prepared by spray drying nisin extract (adjusted to 70% aqueous ethanol) with 2% *w*/*v* zein, 1% *w*/*v* thymol, and glycerol at concentrations 0.05%, 0.1%, 0.5% or 0% (*w*/*v*)	[107]
BB	5- or 6-strain cocktails (A): R2-500, N1-227, N3-031, J1-110, J1-119, R2-502, (B): ATCC 15313, H7762, 2349, 3528, 2422, (C): N3-013, J1-158, J1-169, J1-049, C1-056, (D): DUP-1051D, DUP-1039B, 116-1501-S-4, DUP-1039C, DUP-1059A, (E): 116-110-S-2, DUP-1039E, DUP-1052, ATCC 51775, DUP-1042B	0.36 g/kg caprylic acid + 0.49 g/kg nisin; (0.36 g/kg caprylic acid + 0.40 g/kg nisin − tested with C, D and E cocktails only)	Antimicrobials were added during cheese manufacturing	[108]
BC	5-strain cocktail of: DUP-1030A, DUP-1042, DUP-1042C, DUP-1052A, DUP-10142	0.3, 0.6 or 1.2 g/kg trans-cinnamaldehyde; 0.36 g/kg caprylic acid + 0.49 g/kg nisin; 0.3 g/kg trans-cinnamaldehyde + 0.36 g/kg caprylic acid + 0.49 g/kg nisin	Antimicrobials were added during cheese manufacturing	[108]
BD	CECT 4032 (serotype 4b, previously isolated from a case of meningitis)	(30 mM carvacrol or 1 mM caffeic acid or 10 mM coumaric acid or 10 mM ferulic acid or 1 mM vanillic acid) +20 μg/g enterocin AS-48	Antimicrobials were mixed with salad by gently rolling a pipette over a salad bag	[109]
BE	ATCC 7644	20 μg/mL 1,8-cinole; 0.3 μg/mL carvacrool + 10 μg/mL 1,8-cinole; 0.15 μg/mL carvacrool + 5 μg/mL 1,8-cinole ^3^	Effects of 5 min treatment (submerging in solutions) with antimicrobials at 28 °C were measured; vegetables were not stored after treatment;	[65]
BF	ATCC 7644	N/A	-	[65]
BG	LMG 16779 serovar 1/2a	N/A	-	[74]
BH	ATCC 7644 (C3970)	0.03% (*v*/*v*) citral ^3^	Effects of 60 s subjecting into antimicrobial solutions were measured; samples were not stored after treatment	[69]
BI	ATCC 7644 (C3970)	N/A	Samples were not in contact with solutions, but with antimicrobial vapors	[69]
BJ	3- strain cocktail of: ATCC 15313, H7962, NADC 2045 (Scott A)	Various combinations of carvacrol (1.6–18 ppm) + grapefruit seed extract (0.64–7.36) + nisin (1.6–18.4) ^3^	90 mL of an antibacterial solution was added to 10 g samples	[41]
BK	MTCC No. 1143	500 μg/mL caffeic acid; 500 μg/mL p-Coumaric acid; 500 μg/mL hydroxyl phenyllactic acid; 500 μg/mL tranc sinnamic acid	Apples were immersed in the solutions and then inoculated by dipping in bacterial suspensions	[56]
BL	5-strain cocktail of: Scott A (ATCC), 19115 (ATCC), 101, 1, Presque-598	2% carvacrol ^3^	Washing treatments were applied to rind plugs at 3 levels of temperature (25–65 °C) and at 3 levels of time (exact values depended on temperature); only 65 °C for 5 min washing results are presented in the table as it was the most effective	[110]
BM	5-strain cocktail of Scott A (ATCC), 19115 (ATCC), 101, 1, Presque-598	N/A	Chitosan-based coating treatment was applied to surface-inoculated cantaloupes	[110]
BN	STCC4031	0.4, 0.8 or 1.2 mmol/L of carvacrol and cymene combined	-	[52]

^1^ only studies incorporating NPDAs are included; ^2^ N/A—not applied; ^3^ concentration refers to dipping or coating solution or sauce in which the food product was immersed.
